# The mediating effect of self-efficacy on career aspiration and organizational support with subjective career success among Malaysian women managers during the Covid-19 pandemic

**DOI:** 10.3389/fsoc.2022.802090

**Published:** 2022-08-22

**Authors:** Siti Raba'ah Hamzah, Siti Nur Syuhada Musa, Norhazlina Mohamad

**Affiliations:** ^1^Faculty of Educational Studies, Universiti Putra Malaysia, Serdang, Malaysia; ^2^Department of Academic Affairs, Universiti Teknologi Mara, Melaka, Malaysia

**Keywords:** subjective career success, women manager, career aspiration, occupational self-efficacy, perceived organizational support, COVID-19 pandemic

## Abstract

The lives and jobs of many people have been negatively affected by the Covid-19 pandemic. Lockdowns to curb the pandemic have resulted in many people having to work from home. The question that arises is whether women's jobs are more vulnerable to the crisis. In this regard, it would be useful to understand the factors that affect career success, specifically that of Malaysian women managers. The present study sought to examine the impact of career aspiration and organizational support on subjective career success, as well the mediating role of self-efficacy in these relationships. The participants comprised 146 Malaysian women managers who had completed an occupational self-efficacy scale, career aspiration scale, as well as perceived organizational support and subjective career success inventory. The results of multiple linear regression indicated that those with high levels of career aspiration and perceived organizational support were positively associated with subjective career success. In this connection, self-efficacy mediated both relationships. The findings provided a better understanding of women managers' perception of career success in the service sector.

## Introduction

The Covid-19 pandemic has wreaked havoc in many economies, resulting in not only the temporary disappearance of some occupations and dramatic growth in others, but also in changes in the status of some occupations and their value proposition (Kramer and Kramer, 2020), causing both employers and employees to seek alternative work arrangements (Vyas and Butakhieo, 2020). To curb the spread of the virus, most workers have been told to work from home (WFH). A study by Baert et al. (2020) on the impact of the Covid-19 crisis on career outcomes and career aspirations revealed that many employees are in danger of losing their jobs and missing out on promotions. Autin et al. (2020) identify four emerging impacts of the pandemic, viz. unemployment, worker mental health, work-family interface, and employment disparities. It is important that career development professionals examine these impacts so that they can successfully respond to policies and practices to reduce the severity of the impacts. A recent study by Chauhan et al. (2021) revealed that family responsibilities, mentoring and perceived organizational support variables significantly impacted the perceived career success of women; this finding is a wake-up call for women executives to overcome these impediments so as to achieve successful career advancement. Findings from another study showed that perceived organizational support played a positive moderating role in the relationship between proactive career behavior and subjective career success (Agrawal and Singh, 2022). Career success is defined as an accomplishment and positive work-related outcome associated with work experiences over time and the corresponding career goal-setting across the lifespan (Gunz and Heslin, 2005; Hupkens et al., 2021) Much has been written about the definition of career success from two aspects, viz. objective success and subjective success (Heslin, 2005; Gunz and Mayrhofer, 2011; Spurk et al., 2018; Chen et al., 2021). It is generally accepted that objective career success is the measure of how people are perceived by others in relation to their achievements career-wise, while subjective career success is evaluated by how one feels about his or her own degree of career success (Abele and Spurk, 2009). Objective career success indicators include status and rank (hierarchical position), material success (wealth, property, earning capacity), social reputation and regard, prestige, influence, knowledge and skills, friendships, network connections, health and wellbeing (Heslin, 2005; Ng et al., 2005; Ng and Feldman, 2014).

Theories and research on career-related interests and choices (Lent et al., 1994) are of great relevance to the understanding of employees' responses and reactions to the Covid-19 pandemic with regard to career development. In the present study, the Social Cognitive Career Theory (SCCT) by Lent et al. (1994), which is anchored in the general Social Cognitive theory (Bandura, 1986, 2001), was used for the theoretical framework. The SCCT seeks to explain how career success is obtained, and incorporates a variety of factors including interests, abilities, values, and environmental factors. SCCT emphasizes the individual capacity to direct own behavior. The theory helps to explain the individual's decisions on career interest, goals, and actions vis-à-vis the attainment of the desired level of performance that consists of two main aspects, viz. the objective and subjective dimensions. SCCT also examines personal, environmental, and behavioral variables in complex reciprocal linkages. According to Bandura's general Social Cognitive theory (1986), personal and behavioral variables, as well as the environment, affect career performance. In this study, the individual factor is represented by self-efficacy, the behavioral factor by career aspiration, while the environmental factor refers to perceived organizational support. All these factors are believed to impact career success in three interlocking processes that determine performance attainment, either in the objective or subjective measurement dimension.

Indeed, the Covid-19 pandemic has brought about drastic changes to employment worldwide, with female employees believed to be more adversely affected. There are more challenges for career women (Carli, 2020); they have to cope with flexibility at work as well as housework, caregiving burdens and fears of negative performance evaluation (Thomas et al., 2020). Women are reducing more paid work hours than men and increasing the division regarding the cognitive level of work (Czymara et al., 2020); women also face higher risks of job and income loss as well as increased risks of violence, exploitation, abuse or harassment during times of crisis and quarantine (Mittal and Singh, 2020). Married women employees who have to work from home need also to look after their children and do household chores while attending to office work. According to a recent study, there are emergent changes in work practices and changes for workers, highlighting the impact of the Covid-19 pandemic (Kniffin et al., 2021). Research on career success has focused mainly on engineers, health care workers and financial professionals, while little attention has been paid to women managers during the Covid-19 pandemic. To the best of our knowledge, no study has been conducted on the career success of women managers during the pandemic. Based on the study background, what is the relationship between career aspiration and organizational support with career success among women managers during the COVID-19 pandemic and to what extent does self-efficacy mediate this relationship?

### Career success

Subjective career success indicators include pride in one's achievement, intrinsic job satisfaction, self-worth, commitment to work role or institution, fulfilling relationships, and moral satisfaction (Ng and Feldman, 2014; Smale et al., 2019). However, there have been only a small number of studies on career success literature associated with predictors of career success that integrate the three main approaches (viz. the personal and the behavioral aspects, and the environment) simultaneously in one study. Some of the studies focus on only one or two dimensions such as behavioral approaches (Smale et al., 2019), individual approaches (Rigotti et al., 2018), and individual or organizational approaches such as employee workplace (Callanan, 2003; Spurk et al., 2021). Empirical studies on career success show that men and women perceive career success in different dimensions; women tend to place more emphasis on the importance of balance and relationships, which is a subjective dimension, while men are inclined to focus on material success, which is more toward the objective dimension of career success (Mayrhofer et al., 2008; Afiouni and Karam, 2014; Hartman and Barber, 2020). However, there is a paucity of research on women's career progress as most studies based on the career theory do not specifically show integral gender differences in the measurement dimension related to career advancement (Calinaud et al., 2021).

In this study, the focus was on factors that contributed to the subjective career success of women managers in a Malaysian public university. These women were considered as an under-researched group of employees even though their contributions were very significant and crucial to the development and success of higher learning institutions. Malaysian women managers constituted half of the total managers responsible for the effective operationalisation and administration of many public universities. University managers, also known as “professional staff,” represented 50 percent of non-academic staff that facilitated the organization's operations (Gander et al., 2019). In this study, we integrated three main approaches, namely individual, behavioral, and structural in predicting and explaining career success. The aim was to identify issues that limited or enhanced women managers in achieving career success during the Covid-19 pandemic. A better understanding of career success among women managers would help the development of a career intervention model, improve organizational HRD strategies, and formulate policies and programmes that focus on gender differences in the measurement and definition of career success. The aim of this study was to investigate the mediating effect of self-efficacy on career aspiration and organizational support with subjective career success among Malaysian women managers during the Covid-19 pandemic.

### Career aspiration and career success

Career aspiration reflects the urge to step forward in one's career (Strauss et al., 2012). It acts as a catalyst for spearheading career growth, helping the individual strive toward fulfilling career-related goals (Yun and Min, 2015). Career aspiration also reflects the desire to seek opportunities pertaining to leadership, training and managing others, and furthering one's education (Hartman and Barber, 2020). Indeed, career aspiration is a combination of push factors of the individual, helping him or her give full commitment and attention to anything that will help achieve career success. According to some studies on career aspiration (e.g., Datta and Agarwal, 2017), women's career aspiration is reported to be similar as that of men. However, other researchers argue that men and women have different career aspirations, and that their career aspirations change over time (Yun and Min, 2015). Women with high career aspiration are predicted to show humanistic attitudes related to feminine roles; they have high levels of confidence in playing multiple roles, e.g., as an individuals, part of families and the supervisors in an organization (Kang and Kaur, 2020). In short, career aspiration drives the individual toward desiring excellence in job performance and career outcometo achieve career goals and the high career aspiration leading to high career development opportunities in the organization (Mohd Rasdi et al., 2012; Sharma and Srivastava, 2020). As mentioned above, women's perception of success tends to lean more toward the subjective dimension. Thus, we can expect women's career aspiration to have a positive relationship with subjective career success.

Hypothesis 1: Career aspiration significantly correlates with subjective career success.

### Perceived organizational support and career success

High perceived organizational support (POS) reflects the company's high degree of commitment to its employees; it is characterized over the longer term by trust, support, and respect and care (Shanock and Eisenberger, 2006). When employees perceive that they have organizational support, they expect their company will reward them if they put in greater effort to achieve organizational objectives. This line of thinking is supported by the social exchange theory (Blau, 2017), which posits that when employees perceive that their organization values their contribution to the workplace and cares about their wellbeing, they are more likely to feel obligated to engage in behaviors that are beneficial to their organization. Empirical evidence shows that POS is positively related to positive attitudes and behaviors at the workplace, such as employee performance (Li et al., 2019), work engagement (Imran et al., 2020), affective commitment (Nazir et al., 2018), psychological empowerment and job satisfaction (Maan et al., 2020). Other studies reveal that POS decreases not only burnout and turnover intention of employees (Wang and Wang, 2020) but also work-family conflict (Wattoo et al., 2018). Generally, therefore, POS creates a positive work environment for employees. Nevertheless, the influence of POS on the career success of women managers during the COVID-19 pandemic needs to be examined. Hence, in the current study, we investigated the possibility that perceived organizational support would increase career success despite the pandemic. Additionally, we examined whether the relationship between perceived organizational support and subjective career success was mediated by women managers' self-efficacy. Given the positive effect of POS on employee commitment and job satisfaction, it would be logical to suggest that perceived organizational support is related to career success as well. Nonetheless, as noted earlier, women generally regard career success more from the subjective viewpoint. On the basis of the above, the following hypothesis is proposed.

Hypothesis 2: Perceived organizational support significantly correlates with career success.

### Mediating effect of self-efficacy

Self-efficacy is the individual's perception of his or her ability to successfully implement certain tasks. At the workplace, it refers to how confident individuals feel about successfully carrying out their responsibilities. Self-efficacy has been found to be positively related to subjective career success (Riordan and Louw-Potgieter, 2011). In line with SCCT, self-efficacy represents an individual's confidence in his or her ability to accomplish specific tasks (Hackett and Betz, 1981). A study on the effect of gender on occupational self-efficacy has found that both men and women with high occupational self-efficacy set their own path for career advancement (Hartman and Barber, 2020). It is important to note that individuals with high occupational self-efficacy have considerable control over their career outcome, and are able to enhance their career self-management, thus positively impacting career goals and career success (Ballout, 2009). Furthermore, a study on gender differences of self-efficacy (Dan et al., 2018) shows that women with high self-efficacy set challenging goals, sustain high commitment despite experiencing disappointment, increase and maintain efforts to cope with any work-related failure. As a result, they become very confident in accomplishing their tasks, and this increases their chances of career success. On the other hand, women with negative beliefs about their ability or women with low occupational self-efficacy are unwilling to take risks, do not desire to be highly visible at the workplace, and are negatively self-present (Bandura and Locke, 2003).

According to Abele and Spurk (2009), occupational self-efficacy has a positive impact on both objective and subjective career success. Moreover, self-efficacy mediates innovative behavior and career success (Dan et al., 2018). It is a predictor of career success and performance effectiveness (Ballout, 2009); it helps the individual achieve high job performance and job satisfaction (Hartman and Barber, 2020). Studies show that successful professional women have relatively high self-efficacy (Duffy et al., 2006; Mohd Rasdi et al., 2011). Thus, self-efficacy is expected to have a positive effect on subjective career success.

Hypothesis 3: Self efficacy mediates the relationship between career aspiration and subjective career success.
Hypothesis 4: Self efficacy mediates the relationship between perceived organizational support and subjective career success.

#### Research framework

**Figure 1:**
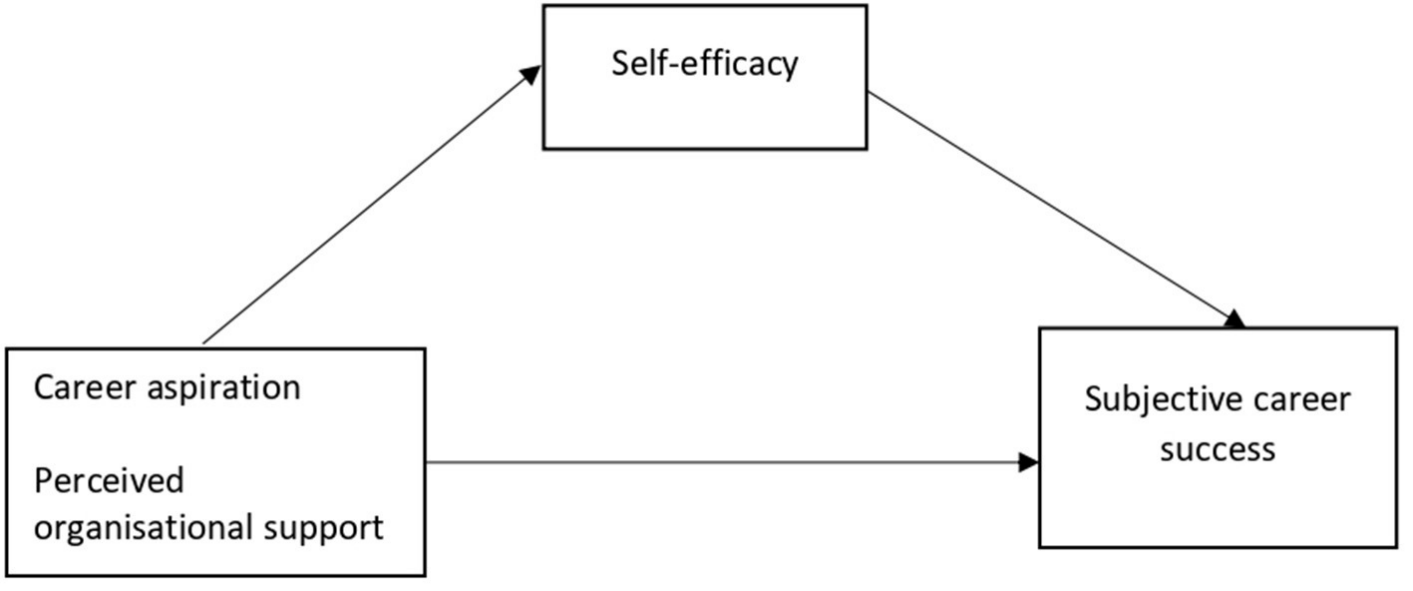
Framework of the study showing the independent variables, the mediator, and the dependent variable.

As illustrated in Figure 1 which depicts the theoretical framework of this study, the aim was to investigate the effects of both individual and organizational factors in influencing subjective career success of Malaysian women managers, with self-efficacy as a mediator. Two models were elaborated on. In the first one, it was hypothesized that self-efficacy mediated the association between career aspiration and subjective career success. In accordance with the inspection procedures of the mediator effect (Baron and Kenny, 1986), we built a simple mediation model (model 4) of the 3.5 macro in SPSS developed by Hayes (2017) to verify the mediating effect of self-efficacy:

Step 1: Career aspiration (X) is associated with subjective career success (Y);
Step 2: Career aspiration (X) is related to self-efficacy, the mediator variable (M);
Step 3: Self-efficacy (M) influences subjective career success (Y).

To determine whether self-efficacy mediated the relationship between career aspiration and subjective career success, we analyzed the effect of career aspiration on subjective career success, while controlling for self-efficacy.

Next, using a second hypothesized model, we examined the contribution of self-efficacy in mediating the relationship between perceived organizational support (POS) and subjective career success. Specifically, it was assumed that:

Step 1: POS (X) is associated with subjective career success (Y);
Step 2: POS (X) is related to self-efficacy, the mediator variable (M),
Step 3: Self-efficacy (M) influences subjective career success (Y).

This procedure was used to assess if self-efficacy mediated the relationship between perceived organizational support (POS) and subjective career success through the analysis of the effect of POS on subjective career success, while controlling for the mediator.

## Methods

### Participants and procedures

To test our proposed model, we applied a quantitative approach to examine the mediating effect of self-efficacy on career aspiration and POS in determining the degree of subjective career success. The target participants for our empirical study were women managers of a large public university in Malaysia. A cross-sectional survey method was used to collect the data. This study employed a self-administered online survey to meet the new norms of research practice as necessitated by the unprecedented pandemic. The data were gathered through an electronic survey questionnaire created with Google Forms; the link was distributed to participants *via* their organization's email address. Prior to conducting this study, ethical approval was granted by the ethics committee of the university (JKEUPM-2021-783). In the questionnaire to collect the data, the authors informed the respondents that the information gathered would be confidential and that it would be used only for research purposes. The data were collected from July 2020 until February 2021. A total of 146 questionnaires were used as the source of final data for statistical analysis, taking into consideration the completeness, readability, and consistency of the responses.

The 146 women managers who participated in the study were aged 20 years and above. Participants aged between 31 and 40 years constituted more than half of the total sample (58.2%). It can be concluded that most of the women managers who participated in this study were at the mid-career stage, i.e., at the stage of career growth, establishment, and maintenance. Mid-career managers generally adopt a more stable emotional and psychological approach when dealing with matters pertaining to job undertaking. The respondents were on different levels of service schemes, viz. Administration and Support (56.8%), Social (21.9%), Financial (12.3%), Engineering (4.8%) and Information System (4.1%). Regarding grade of position, the majority (83.6%) of the respondents held senior positions. The demographic profile on job tenure showed that most respondents (73.2%) had worked between 11 and 20 years; 15.8% had worked >10 years; only 11.0% had worked 21 and more years. The data also revealed that 84.1% of the respondents were married and 15.9% were single.

### Measures

#### The career aspiration scale

The Career Aspiration Scale assesses the element of achievement aspiration, educational aspiration, and leadership (Gregor and O'Brien, 2015). To assess career aspiration, we employed the Career Aspiration Scale (Gray and O'Brien, 2007) that comprises 8 items. Sample item: “I aspire to have my contributions at work recognized by my employer”. The item was scored on a five-point Likert scale ranging from “Untrue of me” (1) to “True of me” (5). The Cronbach's Alpha of the scale has previously been found to be high (α = 0.77) (Strauss et al., 2012). In this study, the Cronbach's Alpha for internal consistency coefficient was calculated as 0.880 for the total score.

#### The perceived organizational support scale

Perceived organizational support was measured using a short version of the survey of Perceived Organizational Support (Eisenberger et al., 1986). Sample item: “*My organization really cares about my wellbeing.”* Each item was rated on a five-point Likert-like scale ranging from “strongly disagree” (1) to “strongly agree” (5). The scale achieved high reliability: α = 0.97 (Neves and Eisenberger, 2014). In this study, the internal consistency coefficient was found to be 0.900 for the total score.

#### The subjective career success inventory

Subjective career success was measured in eight dimensions, viz. (i) authenticity – shaping the direction of one's career according to personal needs and preferences, (ii) growth – growing one's career through the development of new knowledge and skills, (iii) influence – having an impact on others within the organization and on the organization itself, (iv) meaningful work – engaging in work that is personally or socially valued, (v) personal life – having a career that positively impacts life outside of work, (vi) quality of work – producing a high quality product or providing high quality service, (vii) recognition – being formally or informally acknowledged for your work by others, and (viii) career satisfaction sense – positive feelings toward one's career in general. The instrument, known as the Subjective Career Success Inventory (SCSI), consisted of 24 items adapted from Shockley et al. (2015). Sample item: “*The organizations I worked for have recognized me as a good performer.”* It employed a 5-point Likert scale that ranged from 1 = strongly disagree to 5 = strongly agree. The stem for each item starts with “Considering my career as a whole…”. The instrument reported high reliability based on its pilot testing, with α = 0.883.

#### The self-efficacy scale

Self-efficacy was operationalized in this study as occupational self-efficacy. It refers to one's belief in one's own ability and competence to perform and execute behavior relevant to one's occupation and to make judgments about the consequences of successfully performing specific work-related tasks (Hartman and Barber, 2020). To measure self-efficacy in this study, the Occupational Self-efficacy Scale (OCCSEFF) by Schyns and Von Collani (2002) was employed. Sample item: “*I can remain calm when facing difficulties in my job because I can rely on my abilities*.” The scale consisted of eight items and each item was measured on a six-point Likert scale (1 = Not at all true, 2 = Slightly true of me, 3 = Moderately true of me, 4 = True of me, 5 = Very true of me and 6 = Completely true of me. The internal consistency coefficient calculated for the reliability of OCCSEFF for this study was 0.84.

## Data analysis

IBM SPSS Version 25.0 and its plug-in PROCESS macro version 3.4 were used for the analysis of data. Given that there might be a common method variance problem attributed to both subjects' self-reports and similarities of measurement facets between constructs, the Harman single factor analysis method was used to test the common method bias (Podsakoff and Organ, 1986; Fuller et al., 2016). Descriptive statistics for all the analyzed variables to measure the mean and standard deviation. To answer the research question, Pearson's correlations were used to measure the relationship and multiple linear regression analysis were used. The SPSS macro-programme PROCESS v. 3.4 (Hayes, 2018) was employed to test the hypothesized mediation models. The mediation analysis is a regression-based approach that can investigate how and if an independent variable (X) exerts an effect on a dependent variable, postulating the impact of one or more intervening variables (M) positioned between X and Y; in other words, M could be considered as the means by which X has an influence on Y (Hayes, 2018). Therefore, a first single mediation (Model 4) was created to analyse the effect of self-efficacy on the association between career aspiration and subjective career success. Next, the second model analyzed the effect of self-efficacy on the association between perceived organizational support and subjective career success using the mediation model (Model 4). Finally, the indirect effects were estimated following Preacher and Hayes (2004) recommendation of bootstrapping. The 95% bootstrap confidence interval (based on 5000 resamples) was used and all variables were standardized in the analysis. Hayes (2018) emphasizes the usefulness and power of the bootstrapping procedure in mediation analyses as it indicates the significance of the indirect effect when zero is not included in the confidence interval (CI).

## Results

The means, standard deviations, internal consistencies, and correlations were computed for the study variables, as reported in Table 1. Internal consistency (Cronbach's alpha) for all the variables ranged between α = 0.830 and α = 0.900. All significant relationships between the variables were in the expected direction. Career aspiration was found to be positively and significantly correlated with subjective career success (*r* = 0.560, *p* < 0.01). According to Hypothesis 1, subjective career success was positively associated to career aspiration in this sample of women managers. Next, perceived organizational support was positively related to subjective career success (*r* = 0.574, *p* < 0.01). Thus, Hypothesis 2 was accepted. Self-efficacy was also found to be positively and significantly correlated with subjective career success (*r* = 0.435, *p* < 0.01). Hence, high scores on career aspiration, perceived organizational support, and self-efficacy predicted a strong association with subjective career success of the women managers.

**Table 1:** Means, standard deviations, Cronbach alpha, and correlations of main variables.

				**Range**		**Inter-correlations**			
**Variable**	**α**	**M**	**SD**	**Min**	**Max**	**1**	**2**	**3**	**4**
1. SCS	0.883	4.045	0.480	2.67	5.00	1			
2. Self-efficacy	0.830	4.714	0.682	3.00	6.00	0.435^**^	1		
3. Career aspiration	0.880	4.088	0.558	2.75	5.00	0.560^**^	0.482^**^	1	
4. POS	0.900	3.527	0.532	2.00	5.00	0.574^**^	0.276^**^	0.363^**^	1

** P < 0.01; POS, perceived organizational support; SCS, Subjective career success.

The data were further analyzed using the multiple regression analysis with the forced enter method (Table 2) to determine the contributions of career aspiration, perceived organizational support, and self-efficacy to variations in subjective career success among women managers. It was found that career aspiration (β = 0.334, *p* = 0.000), perceived organizational support (POS) (β = 0.408, *p* = 0.000) and self-efficacy (β = 0.162, *p* = 0.020) significantly influenced subjective career success of the women managers who participated in this study [*F*(3, 142) = 45.845, *p* < 0.001]. The largest beta coefficient was 0.408 for POS, making it the strongest contribution in explaining subjective career success. The coefficient of determination, *R*^2^ = 0.492, suggested that 49% of the variance in subjective career success among women managers was explained by the three independent variables, viz. career aspiration, perceived organizational support, and self-efficacy.

**Table 2:** Multiple linear regression for independent variables on subjective career success (*n* = 146).

**Variable**	**Unstandardized**		**Std. Beta**	***t***	***p***
	**Beta**				
	**β**	**Std. error**			
Career aspiration	0.287	0.061	0.334	0.4704	0.000
POS	0.368	0.058	0.408	6.314	0.000
Self-efficacy	0.114	0.048	0.162	2.350	0.020

P < 0.001, β, standardized regression coefficient; t-value, test statistics of β; R = 0.701, R^2^ = 0.492, Adjusted R^2^ = 0.481; POS, Perceived organizational support.

### Mediation analysis

The PROCESS plug-in (Hayes, 2018) was used to perform the mediation analysis, with career aspiration and perceived organizational support as independent variables, subjective career success as a dependent variable, and self-efficacy as a mediation variable (Model 4). First, the variables were standardized. Controlling for the mediating role of self-efficacy on the relationship between career aspiration and perceived organizational support in relation to subjective career success, both were tested using Model 4 of the plug-in PROCESS 3.5 macro in SPSS developed by Hayes (2017). Table 3 shows the first mediation analysis results which indicated that career aspiration directly and positively predicted subjective career success (β = 0.392, *t* = 5.928, *p* < 0.001). In addition, career aspiration positively predicted individuals' levels of self-efficacy (β = 0.589, *t* = 6.609, *p* < 0.001). Self-efficacy had a significant predictive effect on subjective career success (β = 0.151, *t* = 2.797, *p* < 0.001). See Table 3 for the goodness of fit and significance of outcomes and predictors in the tests for the mediating effects of self-efficacy. For the analysis of statistical power, the *R*^2^s in our F-test were 0.232 (self-efficacy as the outcome) and 0.3494 (subjective career success as the outcome). According to the standard for *R*^2^ value proposed by Cohen (2013), the statistical power for career aspiration and self-efficacy relationship was large effect size (*R*^2^ = 0.350) and statistical power for career aspiration, self-efficacy and subjective career success relationship was medium effect size.

**Table 3:** Testing the mediating effects of self-efficacy.

**Regression equation**		**Goodness of** **Fit**		**Significance**		
**Outcome**	**Predictor**	***R^2^***	***F***	**β**	***se***	***t***
**First variable**						
Self-efficacy		0.232	42.628			
	Career aspiration			0.589	0.089	6.605
SCS		0.349	38.394			
	Career aspiration			0.392	0.062	5.928^**^
	Self-efficacy			0.151	0.054	2.797^**^
**Second variable**						
Self-efficacy		0.076	11.815			
	POS			0.353	0.102	3.446
SCS		0.412	50.279			
	POS			0.443	0.060	7.374
	Self-efficacy			0.210	0.046	4.495

** P < 0.001; SCS, Subjective career success; POS, Perceived organizational support.

Next, for the second mediation analysis, the results showed that perceived organizational support directly and positively predicted subjective career success (β = 0.443, *t* = 7.374, *p* < 0.001). In addition, perceived organizational support positively predicted women managers' levels of self-efficacy (β = 0.353, *t* = 3.446, *p* < 0.001) that had a significant predictive effect on subjective career success (β = 0.210, *t* = 4.495, *p* < 0.001). For the analysis of statistical power, the *R*^2^s in our F-test were 0.076 (self-efficacy as the outcome) and 0.412 (subjective career success as the outcome). According to the standard for *R*^2^ value proposed by Cohen (2013), the statistical power for POS and self-efficacy relationship factor was small effect size (*R*^2^ = 0.02) and statistical power for POS, self-efficacy and subjective career success relationship was large effect size.

The significance of the mediating effect was tested with a bootstrap method in the sampling process. PROCESS can construct bias-corrected percentile and Monte Carlo Confidence Interval (CI) for indirect effects (Hayes, 2017). The determination of mediation effect is based on “zero” (0) value location in confidence interval (CI) (Hayes, 2017), if CI does not contain the “zero” (0) value, it means the indirect or mediation effect is statistically significant.

Table 4 shows that the bias-corrected 95% percentile of CI (β = 0.089, CI = 0.017, 0.183) for career aspirations did not include a zero value. These findings hence showed that the indirect effect of career aspiration on subjective career success through the mediator (self-efficacy) was statistically significant, thereby supporting Hypothesis 3. The total effect, direct effect, and total indirect effect for the first mediation analysis were 0.481, 0.392, and 0.089, respectively. The direct effect of career aspiration on subjective career success was 0.392, accounting for 81% of the total effect and the total indirect effect “career aspiration → self-efficacy → subjective career success” accounted for 19% of the total effect.

**Table 4:** Bootstrap analysis of mediating effects of self-efficacy.

				**Bootstrapping**	
				**BC percentile**	
				**95% CI**	
**Path**	**(β)**	**SE**	**Percentage of**	**LL**	**UL**
			**total effect**		
**Career aspiration factor**					
Total effect	0.481	0.059	100%	0.364	0.599
Direct effect: CA → SCS	0.392	0.062	81%	0.261	0.523
Indirect effect: CA → SE → SCS	0.089	0.042	19%	0.017	0.183
**Perceived organizational support factor**					
Total effect:	0.518	0.061	100%	0.396	0.639
Direct effect: POS → SCS	0.443	0.060	86%	0.324	0.562
Indirect effect: POS → SE → SCS	0.074	0.028	14%	0.023	0.134

SCS, Subjective career success; CA, Career aspiration; SE, Self-efficacy; POS, Perceived organizational support; BC, Bootstrap confidence; CI, Confidence interval, Indirect effect is significant if 0 value falls outside the lower bound and upper bound of BC Percentile 95% CI.

Next, the bias-corrected 95% percentile of CI (β = 0.074, CI = 0.023, 0.134) mediation analysis of self-efficacy in the relationship between POS and subjective career success also did not include a zero value. These findings thus showed that the indirect effect of POS on subjective career success through the mediator (self-efficacy) was statistically significant, thereby supporting Hypothesis 4. The total effect, direct effect, and total indirect effect were 0.518, 0.443, and 0.074 respectively. The direct effect of POS on subjective career success was 0.518, accounting for 86% of the total effect and the total indirect effect “POS → self-efficacy → subjective career success” accounted for 14% of the total effect.

## Discussion

The present study focused on the relationship between career aspiration and POS in relation to subjective career success among women managers during the COVID-19 pandemic. Based on the context-process-outcome model and the theory of social cognitive career, a mediation model with self-efficacy as a mediating variable was constructed. Using data from an online survey of 146 women managers, we had two main findings. First, there was a significant positive correlation between career aspiration and perceived organizational support in relation to subjective career success. Second, our analysis also indicated that self-efficacy had a mediating role in the relationship between career aspiration and perceived organizational support with regard to subjective career success; this suggests that enhancing positive self-beliefs such as self-efficacy helps to boost career aspiration and improves subjective career success of women managers. This finding is supported by emerging evidence that career success can be attributed to an individual's action to ensure career development (Abramo et al., 2014).

Furthermore, the present study adds to theoretical contributions of occupational self-efficacy, career aspiration, and perceived organizational support in relation to the attainment of career success. The social cognitive career theory (Brown and Lent, 2004) explains self-efficacy as a mediator and its influence on women managers' career success, along with outcome expectancies of career development. The present findings reflected the theory's structure, with evidence that Malaysian women managers' occupational self-efficacy mediated the relationship of career aspirations and perceived organizational support vis-à-vis career success. This outcome extends previous research that shows the impact of self-efficacy and career success of women managers (Schyns and Von Collani, 2002; Ballout, 2009; Hartman and Barber, 2020).

### Contribution of career aspiration, perceived organizational support, and self-efficacy on career success

In summary, the study results highlight the relevance of the variables used to predict career success. By exploring the interrelationships of career aspiration, perceived organizational support, and self-efficacy to variations in subjective career success among women managers, this study seeks to expand research concerning these variables. Our findings show that subjective career success was positively correlated with career aspiration, perceived organizational support, and self-efficacy. Perceived organizational support (POS) was the strongest predictor of subjective career success. This finding implies that as the level of support offered by the organization increases, subjective career success of the employee also increases. In other words, the higher women managers perceive support from their organization, the more likely they will achieve subjective career success. A possible reason for this might be that the employees are concerned about the extent to which the organization values their contributions and cares about their wellbeing, especially during the COVID 19 pandemic. During this period of working from home, many employees experience insecurity and uncertainty about career development. According to Guan et al. (2020), organizations and governments should take the necessary steps to ensure that career development of individuals is continued. Considering the importance of POS, exchange relations based on reciprocity norms are important in helping to reinforce employee interpersonal relationships within organizations (Eisenberger et al., 2001). Next, the cultures of a particular country can have a great influence on the organization's work culture and practices. Malaysia, generally regarded as a collectivist community with collectivist minds (Lau et al., 2017), fosters strong relationships, where every member of a group takes responsibility for other group members and values highly cooperative or helping behavior and extra-role behavior (Hussain et al., 2017). In such a collectivist country, women managers in this study perceive organization support in terms of being appreciated for their contributions. They feel that their wellbeing is cared for because the Malaysian culture is more collectivistic, respectful of hierarchy and elders, relationship-oriented and cooperative rather than competitive (Merriam and Mohamad, 2000). The results of this study are also in consonance with reports from other studies which find that POS significantly predicts subjective career success of service personnel (e.g., Guan et al., 2016; Dose et al., 2019). Moreover, POS signals important cues to employees that they are valued and possess career potential; these cues are then likely to elicit favorable affective reactions, including higher levels of career satisfaction and a stronger sense of career success. This study further confirms the viewpoint that POS is an important factor that impacts subjective career success (Guan et al., 2017; Erogluer et al., 2020), and extends previous findings related to the pandemic situation, indicating that POS during the pandemic period is a critical factor in career success for employees. As mentioned by Guan et al. (2017), to retain their workforce, companies should recognize and understand the perceptions employees have of their career success.

Evidence from research suggests that interventions related to POS could contribute to employees' career development such as career commitment, career values, and more specifically career success. In this respect, Kurtessis et al. (2017) suggest that POS initiates a social exchange process wherein employees feel obligated to help the organization achieve its goals and objectives, and that increased efforts on the organization's behalf will lead to greater rewards. Furthermore, POS also fulfills socio-emotional needs, resulting in greater identification and commitment to the organization, an increased desire to help the organization succeed, and greater psychological wellbeing among the staff. In line with SCCT, these findings support the notion that environment factors such as POS contribute significantly to subjective career success. In line with past research, POS enhances career success because the resources, information, support and such like facilitate task accomplishment; it also provides access to developmental experiences (Seibert et al., 2001; Forret and Dougherty, 2004). According to Erogluer et al. (2020), employees would have a greater sense of value when they know that their colleagues and managers support them. Undoubtedly, POS plays a critical role in employees' subjective career success.

The second important predictor of subjective career success was career aspiration. The findings in this study indicated that career aspiration was positively related with subjective career success. This implies that as the level of aspiration in career of employees increases, subjective career success of the employee also increases. This is probably because when women managers had a genuine interest and desire to advance their careers, they would trigger more positive attitudes to achieve subjective career success. The present finding is similar with that of a study by Otto et al. (2017) where individuals with strong achievement motivation had higher effort levels for achieving success in their careers. The current COVID-19 pandemic has had a pervasive effect, and the past 2 years have witnessed unprecedented changes at the workplace, switching from the conventional work setting to a new norm of teleworking such as “work from home” (WFH), leading to concerns about “*career shock*.” Despite the challenges, the women managers in this study continued to excel in their work and strived toward meeting job requirements. One possible explanation is that women are generally endowed with skills in adaptability and flexibility, and with increased independence/autonomy when teleworking, women are able to strike a balance between work and family time (Lim and Teo, 2000). From the cultural perspective decades ago, women in Malaysia were mainly occupied with house chores, but now they are also key players in the workforce. The Malaysian workforce composition shows that there is increased involvement of women in the nation's labor force (Department of Statistics, 2018). This reflects a higher level of career aspiration among women and commitment to their careers. The current study suggests that the higher the level of women managers' career aspiration, the more likely they would experience subjective career success. This study further confirms the viewpoint that career aspiration is an important factor influencing subjective career success for women (Li and Huang, 2017), and this applies even during the pandemic situation.

### Mediating effect of self-efficacy

The results of the mediation analysis showed that perceived organizational support (POS) and career aspiration during the pandemic impacted subjective career success through self-efficacy. The women managers in this study were found to have high levels of self-efficacy. Such women have high levels of confidence in their ability and competence to perform and execute behaviors relevant to their occupation and make judgments about the consequences of their decisions (Hartman and Barber, 2020). Self-efficacy, one of the important factors in determining career success among women managers, helps them successfully perform tasks that contribute to their career growth and organizational development.

The results also showed that self-efficacy played a mediating role in the relationship between career aspiration and subjective career success. A high-level of self-efficacy is associated with a high level of career aspiration and subjective career success. This study elucidates the main mechanisms underlying career aspiration and career success, and thereby answers the call made by recent researchers for more studies to explain how subjective career success develops (Rossenkhan et al., 2020). Drawing upon SCCT (Lent et al., 1994), we incorporated self-efficacy as a mediator in our conceptual model and then evaluated the respective effects using the bootstrap method in the sampling process. We found that self-efficacy enhanced subjective career success, and in return, through perceptions of success, self-efficacy was strengthened. The results confirmed that self-efficacy served as a significant mediator in the relationship. Thus, women managers who possess higher confidence and sense of self-worth are more likely to create successful outcomes when they have high career aspiration and are able translate it into action at their workplace. Moreover, efficacious people set more challenging goals for themselves and tend to improve their performance further (Stajkovic and Luthans, 1998). Such people have a greater likelihood of rising to the top in their organization. This supports previous studies that examined the positive role of self-efficacy in improving career aspiration and subjective career success (Chughtai, 2018; Rigotti et al., 2018).

Furthermore, another proposed mediating model of self-efficacy was supported by the data in this study. Results showed that POS had a positive effect on career success through self-efficacy. A plausible explanation is that when women managers had a greater level of self-esteem and self-worth, they were more likely to achieve success in career advancement. Also, when they perceived that they had support from their organization, their socio-emotional needs were met and they experienced greater psychological wellbeing. Women managers with high self-efficacy had more confidence in overcoming setbacks; they set higher career goals, and mobilized all useful resources to achieve those goals. Organizational support has also been found to predict increase in one's self-efficacy within organizational settings (Caesens and Stinglhamber, 2014). According to Bandura (1986), self-efficacy beliefs reflect the intrinsic motivation of employees and stimulate them to improve their work performance. Sung and Connor (2017) postulate that efficacious individuals have an earnest desire to succeed in achieving their goals; they may even inspire others to improve their work behaviors. Hence, for women managers, high levels of perceived organizational support and self-efficacy helped strengthen their perception of career success. This supports previous research which has found that organizational support enhances self-efficacy, and in turn, strengthens the effort to achieve subjective career success (Dan et al., 2018).

## Limitations, implications, and recommendations

This study has some limitations. Our objectives focus on examining the mediating effect of self-efficacy on the relationship of subjective career success with career aspiration and perceived organizational support of only Malaysian women managers. This study employed a quantitative research method and used a self-administered questionnaire for collecting data from women managers. Future studies should collect data more comprehensively, selecting women managers from different sectors with qualitative methods of study. As aspiration and self-efficacy are crucial for practitioners, future researchers should include individual factors such as personality. The results from this study will help organizations better understand the predictors of the career success of women employees. Another practical implication is that the findings of this study provide some useful insight into the role of self-efficacy in the relationship of subjective career success with career aspiration and organizational support. Management could provide more organizational support to facilitate the development of successful careers for women employees.

## Conclusions

Organization should tailor the support extended to employees, especially women employees. This study provides further evidence to defend the argument that supportive management helps enhance employees' self-efficacy beliefs. We describe and assess the relationship between career aspiration and career success practices. Our findings indicate that when perceived organizational support and the need for compliments and recognition are met, women managers would develop positive emotional attitudes and strive to achieve organizational goals. In terms of contributions, our study shows that subjective career success of women is positively correlated with career aspiration and perceived organization support, and that this relationship is mediated by self-efficacy. Our findings also provide a better insight for management regarding employees' career success and their commitment to the organization in a non-western context, specifically in a developing county and culturally unique context such as Malaysia.

## Data availability statement

The raw data supporting the conclusions of this article will be made available by the authors, without undue reservation.

## Ethics statement

Ethical review and approval was not required for the study on human participants in accordance with the local legislation and institutional requirements. The patients/participants provided their written informed consent to participate in this study.

## Author contributions

SH and NM contributed to the conception and design of the study. SH, NM, and SM organized the database, performed the statistical analysis, and wrote sections of the manuscript. NM and SH wrote the first draft of the manuscript and SH its English version. All authors contributed to the manuscript revision, read, and approved the submitted version.

## Conflict of interest

The authors declare that the research was conducted in the absence of any commercial or financial relationships that could be construed as a potential conflict of interest.

## Publisher's note

All claims expressed in this article are solely those of the authors and do not necessarily represent those of their affiliated organizations, or those of the publisher, the editors and the reviewers. Any product that may be evaluated in this article, or claim that may be made by its manufacturer, is not guaranteed or endorsed by the publisher.
